# Relationship Between Abuse and Neglect in Childhood and Diabetes in Adulthood: Differential Effects By Sex, National Longitudinal Study of Adolescent Health

**DOI:** 10.5888/pcd12.140434

**Published:** 2015-05-07

**Authors:** Alexis E. Duncan, Wendy F. Auslander, Kathleen K. Bucholz, Darrell L. Hudson, Richard I. Stein, Neil H. White

**Affiliations:** Author Affiliations: Wendy F. Auslander; Kathleen K. Bucholz, Richard I. Stein, Neil H. White, Washington University and Washington University School of Medicine, St Louis, Missouri; Darrell L. Hudson, Washington University, St Louis, Missouri. Dr Duncan is also affiliated with the Center for Diabetes Translational Research of Washington University and the Midwest Alcoholism Research Center and the Department of Psychiatry, Washington University School of Medicine, St Louis, Missouri.

## Abstract

**Introduction:**

Few studies have investigated links between child abuse and neglect and diabetes mellitus in nationally representative samples, and none have explored the role of obesity in the relationship. We sought to determine whether child abuse and neglect were associated with diabetes and if so, whether obesity mediated this relationship in a population-representative sample of young adults.

**Methods:**

We used data from 14,493 participants aged 24 to 34 years from Wave IV of the National Longitudinal Study of Adolescent Health to study associations between self-reported child abuse (sexual, physical, or emotional abuse) and neglect as children and diabetes or prediabetes in young adulthood. We conducted sex-stratified logistic regression analyses to evaluate associations in models before and after the addition of body mass index (BMI) as a covariate.

**Results:**

Although the prevalence of diabetes was similar for men and women (7.0% vs 6.7%), men were more likely than women to have prediabetes (36.3% vs 24.6%; omnibus *P* < .001). Among men, recurrent sexual abuse (≥3 lifetime incidents) was significantly associated with diabetes (OR, 3.66; 95% CI, 1.31–10.24), but not with prediabetes. There was no evidence of mediation by BMI. No forms of child abuse or neglect were associated with diabetes or prediabetes among women.

**Conclusions:**

Recurrent sexual abuse is robustly associated with diabetes in young adult men, independently of other forms of child abuse or neglect and BMI. Future research should explore other potential mechanisms for this association to identify avenues for prevention of diabetes among men who have experienced sexual abuse.

## Introduction

Diabetes mellitus is the seventh leading cause of death in the United States; the disease affects more than 9% of the US population and costs $245 billion per year (1). An additional 86 million Americans aged 20 years or older are estimated to have prediabetes (1). Although 2012 data indicated a leveling off of the prevalence and incidence of diabetes in the population as a whole, increases are still apparent in some subgroups, including young adults aged 20 to 44 years (2), among whom an estimated 6% have diabetes (3). As the public health burden of diabetes continues to rise, efforts to identify risk factors and stem the tide are needed.

Obesity is a major risk factor for diabetes (4); thus, correlates of obesity are likely targets for associations with diabetes. A recent meta-analysis found that obesity was positively associated with sexual, physical, and emotional abuse in childhood (5), and results from the Adverse Childhood Experiences Study showed that the number of adverse experiences (including all forms of abuse and physical neglect) was significantly related to disordered eating, low levels of physical activity, obesity, and diabetes (6,7). Similarly, a 30-year prospective study found significant associations of childhood maltreatment —including neglect and physical, sexual, and emotional abuse — with obesity (7) and elevated hemoglobin A1c (HbA1c) levels, a marker for diabetes (8). One explanation for the relationship between child abuse and neglect and negative health outcomes is that the chronic stress associated with child maltreatment causes detrimental and lasting neurobiologic changes, such as hypothalamic–pituitary–adrenal (HPA) axis dysregulation, that lead to poor health behaviors and outcomes (6, 9, 10). Additionally, child abuse and neglect may result in conflicted relationships, poor self-esteem, and the subsequent adoption of health-risk behaviors (11).

Child maltreatment is common in the United States. It is estimated that 1 in 8 US children will have an officially confirmed maltreatment episode by age 18 (12). Several studies using nationally representative data from the National Longitudinal Study of Adolescent Health (Add Health) have examined associations between child maltreatment and overweight and obesity or weight gain in adolescence and young adulthood (13–17). Add Health is a longitudinal study of a nationally representative sample of US adolescents who were in grades 7 through 12 during the 1994–95 school year. Add Health collects data on adolescents’ social, economic, psychological, and physical well-being and has followed a cohort of adolescents into young adulthood in a series of in-home interviews called “waves.” These studies using Add Health data had varied results. Three found associations only among particular subgroups; however, results were inconsistent. One study found an association between sexual abuse and obesity in men only (13), 1 study observed a relationship between combined instances of sexual and physical abuse and severe obesity only in nonminority women and men (14), and a third study found that supervisory neglect was associated with body mass index (BMI) at the first wave of data collection (Wave I) in women only (15). Two other studies demonstrated associations in the entire Add Health data between co-occurring physical abuse and neglect and obesity at Wave I (16) and between physical abuse and overweight and obesity at the third wave of data collection (Wave III) (17).

To our knowledge, no study has yet examined associations between child maltreatment and diabetes in a nationally representative sample, and results from studies that used nonrepresentative samples were inconclusive. Furthermore, previous studies did not take obesity into account (18), which would be critical given that obesity, which is a risk factor for diabetes, is associated with childhood maltreatment. Therefore, the objective of this study was to examine whether child maltreatment was associated with diabetes and, if so, whether obesity mediated this relationship in a population-representative sample of young adults.

## Methods

This study used restricted-use data from 14,493 (46.1% male) participants in Wave IV of Add Health. We used variables drawn from participant responses to Waves I, III, and IV in-home interviews. At Wave I, conducted during the 1994–95 school year, a nationally representative sample of 20,745 adolescents in grades 7 through 12 completed in-home interviews. Waves III (2001–02) and IV (2008–09) included all Wave I in-home respondents who could be located, yielding a sample of 15,197 adults aged 18 to 28 years at Wave III, and 15,701 aged 24 to 34 at Wave IV. In addition, at Wave IV, researchers measured height and weight and collected blood for DNA and various biomarker analyses, including fasting or nonfasting blood glucose and HbA1c levels. Details regarding Add Health are available elsewhere (19). Because previous investigations have observed sex-specific associations between childhood maltreatment and obesity, analyses were stratified by sex.

### Key variables

We coded diabetes status from Add Health Wave IV biospecimen data and modeled it as a 3-level variable: 1) diabetes (defined as any of the following: HbA1c ≥6.5%, fasting glucose ≥126 mg/dL, nonfasting blood glucose ≥200 mg/dL, self-reported taking antidiabetic medication, and/or report of receiving a diagnosis of diabetes or high blood glucose by a health care provider); 2) prediabetes or impaired glucose tolerance (HbA1c 5.7%–6.4% and/or fasting glucose 100–125 mg/dL); or 3) no diabetes. We did not use nonfasting blood glucose alone for classification of prediabetes because the American Diabetes Association does not provide guidelines for doing so.

We coded child maltreatment variables from Wave III and Wave IV in-home interview questions (Box) regarding how often respondents experienced specific forms of child maltreatment by adult caregivers. Questions had 5 response options, from “this never happened” to “more than 10 times.” Add Health assessed child neglect at Wave III only and emotional abuse at Wave IV only. Although assessments used identical descriptions of childhood sexual abuse and physical abuse at Wave III and Wave IV, questions at Wave III asked about events occurring before the respondent was in 6th grade; Wave IV questions asked about events occurring before age 18. In both interviews, an additional question about the respondent’s age when the event first occurred followed positive responses. Because the focus of the current study was child abuse and neglect, we coded events reported at Wave IV as positive only if they first happened before age 12. To distinguish recurrent abuse from abuse that occurred only once or twice, we operationalized each type of maltreatment as a 3-level variable: 3 or more times, 1 to 2 times, or never. These cutpoints coincided with the median number of incidents for respondents who had ever experienced physical abuse, sexual abuse, or neglect.

**Box. Questions Used To Assess Childhood Physical Abuse, Sexual Abuse, and Neglect in Study Sample (N = 14,493), Wave III and Wave IV In-Home Interviews, National Longitudinal Study of Adolescent Health**

**Wave III interview (by the time you started 6th grade)**

How often had your parents or other adult caregivers left you home alone when an adult should have been with you? (Neglect)

How often had your parents or other adult caregivers not taken care of your basic needs, such as keeping you clean or providing food or clothing? (Neglect)

How often had your parents or other adult caregivers slapped, hit, or kicked you? (Physical abuse)

How often had your parents or other adult caregivers touched you in a sexual way, forced you to touch him or her in a sexual way, or forced your to have sexual relations? (Sexual abuse)

**Wave IV interview (before your 18th birthday) (coded as positive if age reported for first event was <12 years)**

How often did a parent or other adult caregiver say things that really hurt your feelings or made you feel like you were not wanted or loved? How old were you the first time this happened? (Emotional abuse)

How often did a parent or adult caregiver hit you with a fist, kick you, or throw you down on the floor, into a wall, or down stairs? How old were you the first time this happened? (Physical abuse)

How often did a parent or other adult caregiver touch you in a sexual way, force you to touch him or her in a sexual way, or force you to have sexual relations? How old were you the first time this happened? (Sexual abuse)

Body mass index (BMI [kg/m^2^]) was calculated from height and weight measured at Wave IV. We categorized BMI into 5 levels: obese classes III (≥40), II (35.0–39.9), and I (30.0–34.9); overweight (25.0–29.9); and normal weight (<25.0). Because of low numbers, underweight adults (BMI <18.5) were included in the normal-weight category (n = 191; 1.3% of total sample).

We also included covariates that were known to be associated with both childhood maltreatment and diabetes that were not likely to be in the causal pathway between childhood maltreatment and diabetes and were available in the data set. We modeled the 6-category race/ethnicity preconstructed variable from the Wave I data set (ie, white, black, Latino, Asian/Pacific Islander, American Indian/Native American, and other) (13) as a set of indicator variables, with white as the reference category. We dichotomized self-report of highest education attained at Wave IV as receiving versus not receiving a 4-year college degree. We coded financial insecurity in adolescence from the question in the parental interview: “Do you have enough money to pay your bills?” Because 15.0% of respondents did not have parental interview data, we modeled this variable as a set of indicator variables: enough money to pay bills, not enough money to pay bills (the reference category), or parental data missing. Such subjective measures of social status have been identified as strong predictors of health and, for some measures, are more predictive of health than objective measures such as income and education (20,21). Furthermore, without information about household size or region, estimates of income would not be accurate (22). We obtained information on whether respondents had ever smoked daily from the Wave IV interview.

### Data analysis

We analyzed data from 14,493 Add Health Wave IV participants with biomarker data by using survey procedures in Stata version 9.2 (Stata Corp LP) to account for Add Health’s complex survey design, stratifying all analyses by sex. First, we used χ^2^ analyses to assess bivariate associations of the 3-category diabetes dependent variable (ie, diabetes, prediabetes, or no diabetes) with the 4 child maltreatment variables (ie, sexual abuse, physical abuse, neglect, and emotional abuse) and BMI category and other potential covariates (Table 1). Next, we estimated separate multinomial logistic regression models with 3-category diabetes status as the dependent variable (no diabetes as reference category) for each form of child maltreatment, separately in men and women (models 1–4 [Table 2]). We then estimated a model with all 4 forms of child maltreatment as independent variables (Model 5). To this model, we added the following covariates: age, race/ethnicity, college degree, daily smoking, and childhood financial insecurity (Model 6). Finally, we added BMI category to the model (Model 7) and compared the odds ratios (ORs) of Models 6 and 7. In all models, we conducted post-hoc tests to evaluate differences between the ORs for 1 to 2 versus 3 or more childhood maltreatment incidents for each type of maltreatment.

**Table 1:** Characteristics of Study Sample (N = 14,493) by Sex and Diabetes Status, National Longitudinal Study of Adolescent Health, 2001–2002 and 2008–2009

Characteristic^a^	Women				Men			
	Diabetes^b^	Prediabetes^c^	No diabetes	Omnibus *P* Value	Diabetes^b^	Prediabetes^c^	No diabetes	Omnibus *P* Value
**N**	614	2,062	5,138	—	488	2,584	3,607	—
**Age, y. mean (SD)**	28.43 (0.18)	28.44 (0.13)	28.15 (0.12)	.01	28.79 (0.16)	28.51 (0.13)	28.33 (0.13)	.001
**Race/ethnicity (Wave I^d^)**								
White	45.10	52.12	72.76	<.001	42.66	57.37	75.90	<.001
Black	35.60	26.67	10.60		34.09	21.00	8.55	
Latino	13.16	15.57	10.64		16.07	13.70	9.97	
Asian/Pacific Islander	2.31	3.07	3.36		2.13	4.40	2.84	
American Indian/Native American	3.64	1.57	1.71		4.81	2.75	1.54	
Other	0.18	1.01	0.93		0.23	0.78	1.21	
**Has college degree**	20.50	25.19	36.52	<.001	18.90	19.86	30.96	<.001
**Ever smoked daily for 30 days**	38.96	41.02	47.30	.001	46.53	49.57	53.06	.08
**BMI category**								
Underweight/Normal weight (≤24.9 kg/m^2^)	14.45	22.03	43.83	<.001	16.47	24.94	33.66	<.001
Overweight (25.0–29.9 kg/m^2^)	18.31	22.10	25.87		23.92	33.17	36.66	
Obese class I (BMI 30.0–34.9)	21.75	21.14	14.96		23.82	21.84	19.17	
Obese class II (BMI 35.0–39.9)	19.47	15.87	8.57		12.41	11.17	6.69	
Obese class III (BMI ≥40.0)	26.02	18.87	6.83		23.38	8.88	3.82	
**Childhood financial insecurity (from parent–caregiver interview)**								
Yes	16.93	17.07	14.79	.08	19.96	16.79	11.97	<.001
No	63.91	68.76	70.94		62.07	70.53	74.01	
Missing	19.16	14.16	14.27		17.97	12.68	14.02	
**Childhood sexual abuse (Waves^d^ III, IV)**								
≥3 times	5.36	4.26	4.56	.79	4.10	1.30	1.17	.013
1–2 times	3.05	3.80	4.17		3.64	4.45	3.54	
None	91.60	91.94	91.27		92.26	94.26	95.29	
**Childhood physical abuse (Waves^d^ III, IV)**								
≥3 times	13.64	13.56	15.79	.41	15.32	15.77	15.28	.36
1–2 times	13.01	11.52	11.22		12.70	13.94	11.39	
None	73.35	74.92	73.00		71.98	70.30	73.33	
**Childhood emotional abuse**								
≥3 times	15.32	14.59	18.26	.02	9.81	12.38	13.23	.26
1–2 times	5.08	2.74	3.22		2.10	3.07	3.85	
None	79.61	82.67	78.52		88.09	84.55	82.91	
**Neglect (Wave^d^ III)**								
≥3 times	19.03	16.68	16.95	.11	14.80	17.84	17.96	.62
1–2 times	14.94	18.58	15.49		17.05	18.33	17.96	
None	53.62	48.54	53.10		47.47	45.76	43.75	
Missing	12.42	16.20	14.47		20.68	18.07	20.53	

Abbreviations: BMI, body mass index; HbA1c, Hemoglobin A1c; SD, standard deviation; —, not applicable.

a All values are weighted percentages, unless otherwise noted; all variables were assessed at Wave IV unless otherwise noted with wave number(s) in parentheses.

b HbA1c ≥6.5%, fasting glucose ≥126 mg/dL, nonfasting glucose ≥200 mg/dL, self-reported taking antidiabetic medication and/or a positive answer to the question “Has a doctor, nurse or other health care provider ever told you that you have or had high blood sugar or diabetes?”

c HbA1c 5.7%–6.4% and/or fasting glucose 100–125 mg/dL.

d Waves refer to the series of 4 in-home interviews through which Add Health collected data on adolescents’ social, economic, psychological, and physical well-being.

**Table 2:** Characteristics of Study Sample (N = 14,493) by Sex and Wave IV Body Mass Index (BMI) Category, National Longitudinal Study of Adolescent Health, 2001–2002 and 2008–2009^a^

Type and Lifetime Frequency of Maltreatment	Underweight/Normal weight (BMI ≤24.9 kg/m^2^)	Overweight (BMI 25.0–29.9 kg/m^2^)	Obese Class I (BMI 30.0–34.9 kg/m^2^)	Obese Class II (BMI 35.0–39.9 kg/m^2^)	Obese Class III (BMI ≥40.0 kg/m^2^)	Omnibus *P* Value
**Men**						
**Sexual abuse**						
≥3 times	1.35	1.39	1.58	1.45	1.53	.37
1–2 times	3.29	3.46	4.45	3.65	7.35	
None	95.36	95.15	93.96	94.91	91.12	
**Physical abuse**						
≥3 times	15.84	13.87	15.27	22.36	14.19	.012
1–2 times	13.34	11.16	11.59	12.31	17.13	
None	70.82	74.97	73.14	65.33	68.68	
**Emotional abuse**						
≥3 times	13.26	11.37	14.55	13.06	10.96	.40
1–2 times	4.06	3.02	2.85	4.20	3.93	
None	82.68	85.61	82.60	82.74	85.11	
**Neglect**						
≥3 times	17.96	16.53	18.40	17.79	18.83	.83
1–2 times	18.12	17.17	18.67	18.21	19.36	
None	45.48	44.77	43.09	46.49	45.55	
Missing	18.43	21.53	19.84	17.51	16.26	
**Women**						
**Sexual abuse **						
≥3 times	3.66	4.93	6.02	4.72	4.06	.45
1–2 times	4.45	3.84	3.63	3.62	4.26	
None	91.89	91.22	90.36	91.66	91.68	
**Physical abuse **						
≥3 times	14.66	16.82	13.95	13.46	15.63	.04
1–2 times	11.34	10.26	10.30	11.16	16.25	
None	74.00	72.92	75.75	75.38	68.12	
**Emotional abuse **						
≥3 times	17.20	17.96	15.96	14.29	20.34	.37
1–2 times	3.09	3.28	3.45	2.48	3.84	
None	79.71	79.03	80.59	83.23	75.82	
**Neglect**						
≥3 times	16.09	17.30	18.63	15.62	19.42	.76
1–2 times	15.55	15.88	16.83	18.35	15.37	
None	53.95	51.96	49.27	51.85	50.85	
Missing	14.40	15.13	15.28	14.18	14.36	

a All values are weighted percentages unless otherwise noted.

## Results

Although the prevalence of diabetes was similar for men and women (7.0% vs 6.7%), men were more likely than women to have prediabetes (36.3% vs 24.6%; omnibus *P* < .001). Both men and women with diabetes were more likely than men and women without diabetes to have a BMI in the obese range and to be a member of a racial/ethnic minority group and less likely to report having a college degree. Men, but not women, with diabetes were significantly more likely to have a background of childhood financial insecurity. The prevalence of these variables for respondents with prediabetes was generally between the prevalence for those with and without diabetes (Table 1). In both men and women, a history of daily smoking was inversely associated with diabetes; however, these associations were significant only among women (*P* = .001).

Among men, 4.1% with diabetes reported that they had experienced sexual abuse by a caregiver 3 or more times, compared with 1.3% with prediabetes and 1.2% without diabetes (*P* = .013). No other forms of child maltreatment were significantly associated with diabetes among men. Among women, only emotional abuse was associated with the 3-level diabetes status variable overall (*P* = .02), but the relationship was complex. Although women with diabetes had a higher prevalence of 1 to 2 occurrences of emotional abuse than women with prediabetes or women without diabetes, they had a lower prevalence of 3 or more occurrences of emotional abuse (15.3% vs 18.3% for women without diabetes). Childhood physical abuse was significantly associated with BMI category in men (*P* = .012) and in women (*P* = .04); however, there were no significant associations between BMI category and any other forms of childhood maltreatment in men or women (Table 2).

Men who reported experiencing sexual abuse 3 or more times had 3.63 times greater odds of diabetes than men who did not report sexual abuse (95% CI, 1.53–8.62) (Table 3). The magnitude of this association remained similar after adjusting for other forms of child maltreatment and covariates (OR, 3.66; 95% CI, 1.31–10.24). The addition of BMI category to the model slightly increased the magnitude of association between 3 or more incidents of sexual abuse and diabetes (OR, 3.80; 95% CI, 1.48–9.72), an indication that BMI category did not mediate the association; all BMI categories were significantly, positively associated with diabetes and prediabetes. Negative associations between diabetes and both neglect and emotional abuse were observed but were not consistently significant, and no associations were noted for physical abuse. With one exception (infrequent physical abuse in bivariate model only), no other child maltreatment variables were associated with prediabetes in men.

**Table 3:** Results from Multinomial Logistic Regression Models Predicting Diabetes Status for Men and Women (N = 14,493) Participating in the National Longitudinal Study of Adolescent Health, 2001–2002 and 2008–2009

Type and Lifetime Frequency of Maltreatment	Men		Women	
	Diabetes, OR (95% CI)	Prediabetes, OR (95% CI)	Diabetes, OR (95% CI)	Prediabetes, OR (95% CI)
**Model 1: Sexual abuse only**				
≥3 times	3.63 (1.53–8.62)^a^	1.13 (0.61–2.10)	1.17 (0.76–1.82)	0.93 (0.64–1.14)
1–2 times	1.06 (0.47–2.37)^b^	1.27 (0.86–1.88)	0.73 (0.34–1.57)	0.90 (0.62–1.32)
Never	1 [Reference]	1 [Reference]	1 [Reference]	1 [Reference]
**Model 2: Physical abuse only**				
≥3 times	1.02 (0.70–1.49)	1.08 (0.89–1.31)	0.86 (0.59–1.24)	0.84 (0.67–1.05)
1–2 times	1.14 (0.67–1.93)	1.27 (1.01–1.61)	1.15 (0.79–1.69)	1.00 (0.81–1.24)
Never	1 [Reference]	1 [Reference]	1 [Reference]	1 [Reference]
**Model 3: Emotional abuse only**				
≥3 times	0.70 (0.44–1.10)	0.92 (0.71–1.18)	0.83 (0.57–1.20)^b^	0.76 (0.62–0.93)^a^
1–2 times	0.51 (0.24–1.11)	0.78 (0.51–1.19)	1.56 (0.92–2.62)^c^	0.81 (0.52–1.26)
Never	1 [Reference]	1 [Reference]	1 [Reference]	1 [Reference]
**Model 4: Neglect only**				
≥3 times	0.77 (0.49–1.19)	0.96 (0.78–1.18)	1.11 (0.81–1.52)	1.08 (0.87–1.33)
1–2 times	0.88 (0.60–1.28)	0.98 (0.80–1.20)	0.96 (0.67–1.37)	1.31 (1.06–1.63)
Missing	0.93 (0.63–1.38)	0.85 (0.69–1.04)	0.85 (0.63–1.15)	1.22 (0.93–1.61)
Never	1 [Reference]	1 [Reference]	1 [Reference]	1 [Reference]
**Model 5: all forms of maltreatment**				
**Sexual abuse**				
≥3 times	4.26 (1.75–10.37)^a^	1.08 (0.56–2.07)	1.14 (0.69–1.89)	0.95 (0.64–1.40)
1–2 times	1.13 (0.49–2.58)^b^	1.22 (0.78–1.90)	0.83 (0.39–1.78)	0.96 (0.66–1.40)
Never	1 [Reference]	1 [Reference]	1 [Reference]	1 [Reference]
**Physical abuse**				
≥3 times	1.20 (0.80–1.78)	1.10 (0.88–1.37)	0.83 (0.53–1.30)	0.93 (0.72–1.21)
1–2 times	1.19 (0.73–1.94)	1.30 (0.99–1.71)	1.08 (0.74–1.60)	1.00 (0.81–1.24)
Never	1 [Reference]	1 [Reference]	1 [Reference]	1 [Reference]
**Emotional abuse**				
≥3 times	0.65 (0.42–1.00)	0.90 (0.70–1.15)	0.85 (0.57–1.29)^a^	0.77 (0.60–0.97)
1–2 times	0.50 (0.23–1.10)	0.75 (0.49–1.16)	1.56 (0.93–2.64)^b^	0.76 (0.48–1.20)
Never	1 [Reference]	1 [Reference]	1 [Reference]	1 [Reference]
**Neglect**				
≥3 times	0.68 (0.42–1.12)	0.92 (0.73–1.15)	1.09 (0.77–1.55)	1.14 (0.91–1.43)
1–2 times	0.80 (0.53–1.21)	0.90 (0.72–1.12)	0.98 (0.68–1.41)	1.29 (1.04–1.60)
Missing	0.95 (0.62–1.43)	0.89 (0.71–1.11)	0.87 (0.63–1.20)	1.22 (0.95–1.56)
Never	1 [Reference]	1 [Reference]	1 [Reference]	1 [Reference]
**Model 6: all forms of maltreatment and covariates^c^**				
**Sexual abuse**				
≥3 times	3.66 (1.31–10.24)^a^	0.95 (0.50–1.83)	1.04 (0.63–1.72)	0.91 (0.61–1.35)
1–2 times	0.74 (0.33–1.67)^b^	0.98 (0.62–1.55)	0.70 (0.32–1.50)	0.86 (0.59–1.26)
Never	1 [Reference]	1 [Reference]	1 [Reference]	1 [Reference]
**Physical abuse**				
≥3 times	1.11 (0.73–1.70)	1.01 (0.82–1.25)	0.77 (0.50–1.20)	0.88 (0.68–1.14)
1–2 times	1.25 (0.77–2.03)	1.30 (0.99–1.72)	1.02 (0.69–1.51)	0.97 (0.78–1.20)
Never	1 [Reference]	1 [Reference]	1 [Reference]	1 [Reference]
**Emotional abuse**				
≥3 times	0.74 (0.47–1.16)	0.97 (0.75–1.25)	1.02 (0.68–1.54)	0.88 (0.69–1.12)
1–2 times	0.45 (0.20–1.04)	0.73 (0.46–1.16)	1.57 (0.90–2.72)	0.75 (0.47–1.21)
Never	1 [Reference]	1 [Reference]	1 [Reference]	1 [Reference]
**Neglect**				
≥3 times	0.60 (0.37–1.00)	0.87 (0.69–1.09)	1.00 (0.68–1.46)	1.06 (0.84–1.34)
1–2 times	0.79 (0.52–1.22)	0.89 (0.70–1.12)	0.98 (0.67–1.42)	1.29 (1.02–1.63)
Missing	0.74 (0.47–1.16)	0.79 (0.64–0.98)	0.68 (0.49–0.94)	1.02 (0.80–1.29)
Never	1 [Reference]	1 [Reference]	1 [Reference]	1 [Reference]
**Model 7: all forms of maltreatment, covariates^c^ and BMI**				
**Sexual abuse**				
≥3 times	3.80 (1.48–9.72)^a^	0.93 (0.49–1.77)	1.03 (0.62–1.71)	0.90 (0.61–1.32)
1–2 times	0.64 (0.30–1.37)^b^	0.92 (0.58–1.48)	0.71 (0.32–1.59)	0.89 (0.58–1.37)
Never	1 [Reference]	1 [Reference]	1 [Reference]	1 [Reference]
**Physical abuse**				
≥3 times	1.15 (0.76–1.75)	0.99 (0.81–1.22)	0.76 (0.48–1.21)	0.87 (0.66–1.14)
1–2 times	1.19 (0.74–1.91)	1.31 (1.00–1.71)	0.95 (0.66–1.36)	0.92 (0.73–1.14)
Never	1 [Reference]	1 [Reference]	1 [Reference]	1 [Reference]
**Emotional abuse**				
≥3 times	0.70 (0.44–1.09)	0.97 (0.75–1.26)	1.00 (0.67–1.48)	0.86 (0.67–1.09)
1–2 times	0.44 (0.20–0.99)	0.71 (0.45–1.12)	1.67 (0.97–2.86)	0.77 (0.47–1.27)
Never	1 [Reference]	1 [Reference]	1 [Reference]	1 [Reference]
**Neglect**				
≥3 times	0.63 (0.38–1.04)	0.89 (0.70–1.12)	0.95 (0.64–1.43)	1.03 (0.81–1.32)
1–2 times	0.84 (0.53–1.33)	0.88 (0.70–1.12)	0.97 (0.65–1.45)	1.29 (1.02–1.63)
Missing	0.82 (0.54–1.25)	0.81 (0.65–1.01)	0.72 (0.51–1.00)	1.06 (0.83–1.35)
Never	1 [Reference]	1 [Reference]	1 [Reference]	1 [Reference]
**BMI category**				
Obese class III	12.02 (7.21–20.03)	2.98 (1.98–4.50)	9.24 (6.05–14.11)	4.48 (3.42–5.87)
Obese class II	3.80 (2.33–6.19)	2.16 (1.63–2.87)	5.71 (3.40–9.59)	3.27 (2.46–4.34)
Obese class I	2.60 (1.65–4.09)	1.55 (1.22–1.96)	3.56 (2.46–5.75)	2.45 (1.99–3.03)
Overweight	1.35 (0.85–2.16)	1.25 (1.04–1.52)	2.01 (1.26–3.20)	1.67 (1.34–2.10)
Normal weight	1 [Reference]	1 [Reference]	1 [Reference]	1 [Reference]

Abbreviations: BMI, body mass index; CI, confidence interval.

a,b Values with different superscripts are significantly different from one another in pairwise post-hoc tests (*P* < .05).

c Adjusted for age, race/ethnicity, college degree, ever smoked daily, and childhood financial insecurity.

In contrast to men, among women (Table 3) no associations between diabetes and any child maltreatment variable were observed in any of the models. Rather, women experiencing 1 to 2 occurrences of neglect had a greater risk of prediabetes (OR, 1.31; 95% CI, 1.06–1.63) that remained significant and similar in magnitude even after covariates and BMI were added to the model (OR, 1.29; 95% CI, 1.02–1.63). There was an inverse association between repeated emotional abuse and prediabetes; however, this relationship was no longer significant after adding covariates and BMI to the model.

## Discussion

In this population-based sample of young adults, we found that repeated sexual abuse was significantly associated with diabetes among men, even after adjusting for BMI category. In contrast, there were no associations between retrospective self-reports of any form of childhood maltreatment and diabetes among women. There is limited previous research in this area, and findings from other studies of child maltreatment and diabetes have been mixed. Our results are consistent with those reported in a previous Add Health study that used data from Wave III and found a significant association between sexual abuse and obesity in men but not in women (13).

To our knowledge, this is the first study to investigate the possibility that BMI mediates the association between childhood maltreatment and diabetes. BMI category was positively associated with prediabetes and diabetes in these analyses for both women and men; however, it did not serve as a mediator of the relationship between sexual abuse and diabetes in men, as evidenced by the increase in the magnitude of the OR for sexual abuse after adding BMI category to the model. This finding was not unexpected given that we did not observe an association between sexual abuse and obesity in sexual abuse data from Waves III and IV and BMI data from Wave IV (Table 2), contrary to the results of a previous study (13). Although a meta-analysis showed a significant positive association between sexual abuse and obesity, many of the individual studies included in the meta-analysis did not find a significant association, and ORs from these studies ranged from 0.81 to 3.60 (5). This heterogeneity may be due to differences in the operationalization of sexual abuse and to choice of the comparison group for obesity. Furthermore, findings from previous Add Health studies of associations between sexual abuse and obesity and BMI varied and found significant associations only within particular subgroups (13–15). Taken together, this suggests that if there is an association between sexual abuse and obesity and BMI in Add Health, it is not a robust one.

There is a well-established association between childhood adversity and mental and physical health outcomes (8,15,16,24). Our results suggest that sexual abuse may also have a negative effect on the physical health of men, in particular on cardiovascular disease risk. Fuller-Thomson et al (25), in a population-based adult sample, reported significantly elevated odds of myocardial infarction among men, but not women, who were exposed to childhood sexual abuse than among their unexposed counterparts (25). Various explanations have been offered for this link that may be relevant to our results (24). These include that men could be less likely than women to seek treatment following incidents of abuse and that treatment regimens for men may differ from those for women, leading to poorer adaptation of men following the abuse. This poor adaptation may increase men’s psychosocial stress, thereby affecting the HPA stress pathway, making them more vulnerable to adverse cardiovascular events, including precursor outcomes such as diabetes. Further research in this area is needed. The prevalence of officially confirmed childhood sexual abuse and rates of sexual abuse based on retrospective self-report are lower for men than for women (26–28). Thus, efforts to improve the identification of childhood sexual abuse among men and subsequent interventions may not only improve psychosocial outcomes but may also benefit men’s long-term physical health.

This study also may be the first to examine associations between childhood maltreatment and prediabetes; however, some of the results were inconsistent and somewhat counterintuitive. For example, women who reported experiencing 1 to 2 neglect incidents were more likely to develop prediabetes, but the OR for 3 or more incidents was close to 1 and not significant; this was also true for both levels of neglect when predicting diabetes. This finding may be due to chance, particularly given that there was not a significant difference between the ORs for 1 to 2 neglect incidents and 3 or more neglect incidents. The negative association between infrequent emotional abuse and diabetes in men was also unexpected and may possibly reflect limitations of the Add Health child maltreatment assessment. Because the assessment of each form of child maltreatment was limited to 1 or 2 questions, it is difficult to place these results into context. A single-item indicator for child maltreatment events may not be adequate in accurately capturing all cases of abuse and does not allow for its in-depth characterization. In addition, the Add Health assessment of sexual abuse asked only about incidents committed by a family member or adult caregiver. Because many perpetrators of sexual abuse, particularly among male victims, are not relatives or caregivers (24,27), people who experienced sexual abuse perpetrated by persons not family members or caregivers would be false negatives on the sexual abuse variable, causing a bias toward the null hypothesis. Misclassification from retrospective reports of child maltreatment, another potential limitation of the assessment, would also probably yield far more false negatives than false positives. Therefore, the true magnitude of association between childhood sexual abuse and diabetes in men may be greater than we observed.

There were also limitations to our categorization of diabetes. First, we were unable to distinguish between type 1 and type 2 diabetes. It is possible that results may have differed by diabetes type, particularly because risk factors for the two types are different (29); however, given estimates that 90% to 95% of adults with diabetes have type 2 diabetes (3), most people with diabetes in the sample probably had type 2 diabetes. Second, the American Diabetes Association currently defines blood glucose cutoffs for classifying prediabetes solely based on fasting blood glucose, but for most Add Health participants, only nonfasting blood glucose levels were available. Thus, we may have missed prediabetes in some people for whom we did not have fasting blood glucose levels: 1.4% of people classified as not having diabetes (n = 123) had nonfasting blood glucose levels in the 140 to 199 mg/dL range and reported 2 to 7 hours since they last ate or drank; some of these may have had prediabetes. Second, 5.2% of women with diabetes (n = 32), but no men, were coded as such solely because they were on antidiabetes medication; it is possible that some of those women were on metformin because of polycystic ovary syndrome rather than for diabetes. Removal of these women from the analysis, however, did not alter the results (data not shown).

Although results were not uniform, our findings indicate that childhood sexual abuse among men is associated with greater risk of diabetes in adulthood. The magnitude of effect remained robust even when controlling for other forms of child maltreatment and obesity. The prevalence of sexual abuse may be underestimated in this sample. Furthermore, given the potential for interactions between different variables examined here, unobserved factors could have altered different associations of diabetes with childhood abuse and neglect. Nonetheless, these findings from a large, nationally representative sample that used a rigorous approach to defining diabetes are a potentially important step in understanding the relationship between childhood adversities and diabetes and may generate future research efforts to develop new hypotheses and interventions to address factors related to the development of diabetes.
